# *“The way the country has been carved up by researchers”*: ethics and power in north–south public health research

**DOI:** 10.1186/s12939-016-0488-4

**Published:** 2016-12-12

**Authors:** Aisling Walsh, Ruairi Brugha, Elaine Byrne

**Affiliations:** 1 Department of Epidemiology and Public Health Medicine, Royal College of Surgeons in Ireland, Dublin, Ireland; 2 Institute of Leadership, Royal College of Surgeons in Ireland, Dublin, Ireland

**Keywords:** Zambia, Research partnerships, Situated research ethics, Power, Bourdieu

## Abstract

**Background:**

Despite the recognition of power as being central to health research collaborations between high income countries and low and middle income countries, there has been insufficient detailed analysis of power within these partnerships. The politics of research in the global south is often considered outside of the remit of research ethics. This article reports on an analysis of power in north–south public health research, using Zambia as a case study.

**Methods:**

Primary data were collected in 2011/2012, through 53 in-depth interviews with: Zambian researchers (*n =* 20), Zambian national stakeholders (*n =* 8) and northern researchers who had been involved in public health research collaborations involving Zambia and the global north (*n =* 25). Thematic analysis, utilising a situated ethics perspective, was undertaken using *Nvivo 10*.

**Results:**

Most interviewees perceived roles and relationships to be inequitable with power remaining with the north. Concepts from Bourdieu’s theory of Power and Practice highlight new aspects of research ethics:

- Northern and southern researchers perceive that different *habituses* exist, north and south - *habituses* of domination (northern) and subordination (Zambian) in relation to researcher relationships.
- Bourdieu’s *hysteresis* effect provides a possible explanation for why power differentials continue to exist. In some cases, new opportunities have arisen for Zambian researchers; however, they may not immediately recognise and grasp them.
- Bourdieu’s concept of *Capitals* offers an explanation of how diverse resources are used to explain these power imbalances, where northern researchers are often in possession of more economic, symbolic and social capital; while Zambian researchers possess more cultural capital.

**Conclusions:**

Inequities and power imbalances need to be recognised and addressed in research partnerships. A situated ethics approach is central in understanding this relationship in north–south public health research.

## Background

The value of international research collaborations has been well documented [1–5]. A review of the literature on health research between lower and middle income countries (LMIC) and higher income countries^1^ (HIC) - referred to in this paper as the ‘global north and south’ -confirms that power imbalances and inequities exist at each stage of the research process: from funding, to agenda setting, data collection, analysis and research outputs [6–12]. Some of these imbalances are structural (such as funding) [9, 10, 13–16] and some are related to inequitable relationships [11, 17, 18], though both are interlinked.

A north–south dichotomy dominates evaluations of north–south health research.^2^ Inequities and power imbalances between researchers in LMIC and HIC are often considered outside the immediate remit of research ethics, resulting in researchers, funders and institutions ignoring fundamental ethical issues of the politics and inequity of research in LMIC. Recently, there have been calls for consideration of the processes of collaboration between the north and south as ethical concerns [17–25]. This has been termed by some authors a ‘*situated ethics’* of research:

*“We are concerned that issues of ethics should be seen as integral to the whole research process… about researchers interrogating and responding to unequal power relations…”* [20]

This paper reports on an analysis of power in north–south public health and health systems research, specifically a ‘situated ethics’ of research analysis [11, 20, 25]. The focus on research partnerships is through the lens of researchers, who are the people that define, shape and execute such research studies, and include the broader issues of the politics and power of the research process, from agenda setting to capacity building, to authorship; and how research actors and institutions function and interact. For the most part, the literature does not designate these as ethical issues, and most research ethics guidelines [26–28] and published studies of research ethics focus instead on traditional areas such as research ethics review processes [29–33] and informed consent [34–36]. However, we argue that these broader ethical issues are of equal importance.

Despite the recognition of power as being central to health research collaborations, there is limited, if any, detailed analysis of power within north–south health research. The published literature on such collaborations does not provide detailed insight into why power imbalances persist and where discussed are often from self-evaluations or reflections and as such may underplay inequalities in north–south health research. This paper addresses this gap from a situated ethics of research perspective: How does power influence international academic north–south public health research, and why do power imbalances exist and persist? Power for this paper is best defined as *“the production, in and through social relations, of effects that shape the capacities of actors to determine their circumstances and fate* [37]*.* While north–south dichotomies dominate the literature on north–south health research, the purpose of this paper is to highlight that a more nuanced approach to such dichotomies needs to be developed if we are to understand such partnerships.

## Theoretical framework

Concepts from Pierre Bourdieu’s theory of *Power and Practice* [38] provide a useful lens to structure and discuss the situated ethics of research dimensions of public health and health systems research that emerged from this study.

Bourdieu contends that power is at the heart of all social life [39] and hence are also at the heart of research relationships. At the centre of Bourdieu’s analysis is the question: why do social inequalities persist? Bourdieu’s theory [38] conceptualises action as the outcome of a relationship between *Field*, *Habitus, and Capital.* These concepts are useful in exploring the same question of inequalities in research relationships and are therefore examined in more detail here.

### The field

Bourdieu describes society as consisting of a system of fields, each one with its own structure which is semi-autonomous and set within a larger field of power [38]. Fields are structured spaces of dominant and subordinate positions that are organised around specific types of capital or combinations of capital; and where actors struggle to accumulate these different kinds of capital [40]. In the study described in this paper, the arena is the field of international academic north–south public health research. The occupants of positions within a field may be either agents (in this case researchers) or institutions (Universities or research institutions) which are constrained or enabled by the structure of the field [40].

### Habitus

*Habitus* refers to the values and expectations of particular social groups that are acquired as a result of a long-term occupation in a social world [38]. *Habitus* defines what is possible within a certain group, generating a self-fulfilling prophecy [39]. Dominant and subordinate positions must be identified for all the participants in the field.

*Habitus* has moments when it is out of phase, particularly when a field undergoes a transformation that changes its rules. A structural lag can occur in these circumstances, − a ‘*hysteresis effect’* [39] - between aspirations and changing opportunities. The concept of *hysteresis* is particularly useful for explaining northern and southern positions in health research, in particular for explaining why power imbalances exist and persist, i.e. why the *status quo* remains unchanged, despite an impetus for change at the global level, illustrated, for example, in the calls to build capacity for health research leaders in LMIC [41–43].

### Capital

According to Bourdieu, individuals and groups draw upon a variety of resources to maintain and enhance their positions in the social order [39]. The capital that people accumulate defines their social trajectory [44]. Fields are organised around a combination of economic, social, symbolic and cultural capital. Researchers’ accumulation of capital can help us to understand their trajectory and in particular power differentials in the field of north–south health research.

Bourdieu contends that economic capital is *“at the root of all other types of capital”* [38] thereby proposing it as the capital with the most influence. Social capital refers to collaboration between individuals and groups. Bourdieu views social capital from the perspective of producing or reproducing inequality [38] rather than a way to promote equality*.*

Bourdieu asserted that symbolic capital is a resource available to an individual based on prestige and recognition [40]. Scientific capital is classed as a form of symbolic capital based on the prestige of the University they are attached to [44].

Cultural capital is identified by Bourdieu [38] as existing in three different states.

- *Internalised:* refers to dispositions that are internalised by the individual through socialisation and that constitute patterns of understanding, such as northern researcher or southern researcher culture.
- *Objectified:* referring to objects, such as books and scientific instruments that require specialised cultural abilities to use, such as the culture of science, specifically in health research.
- *Institutionalised:* the educational credential system, namely qualifications, degrees or titles [39], such as educational culture and work culture in north–south health research.

According to Bourdieu, cultural capital is a major source of social inequality. He asserted that sharing similar forms of cultural capital with others creates a sense of collective identity and group position [40].

## Methods

### Setting

Zambia was chosen as a suitable single case study [45] that explores the ethics of health research between the global north and the global south. According to the Zambia Forum for Health Research, health research in Zambia is fragmented and underfunded [46]; health research priority setting has been ad hoc [47] and external donors fund up to 90% of health research [48]. Recent years have witnessed attempts to establish a national health research system, including legislation and regulation [49]. In addition two of the authors have undertaken health research in Zambia over a number of years and therefore have knowledge of the context.

### Research design

Qualitative data collection and analysis methods were selected for this study, to understand and discover southern and northern researchers’ interpretations and experiences of being involved in north–south health research collaborations [50, 51]. The lead author undertook 53 interviews (see Table 1). Twenty of these were Zambian researchers, and 8 were national level stakeholders who had been involved in setting up the Zambian health research system. Twenty five northern researchers were included in the sample, 4 of whom were considered to be north–south researchers: 3 of these were northern researchers who had lived in Zambia for a long period of time; and 1 South African researcher who was involved in a multi-country collaboration involving Zambia.^3^ A purposive sampling strategy was employed, which involved selecting participants on the basis of their academic background, gender, roles and career stage/experiences in north–south health research.

**Table 1:** Interviewee attributes

	Northern	Zambian	Total
Geographic location			
North	21	-	21
Zambian	-	28	28
North–south	4	-	4
Sex			
Male	11	18	29
Female	14	10	24
Institutional affiliation			
University	18	14	32
Research institution	3	8	11
Government	-	3	3
NGO	4	3	7
Career level			
Junior-middle level	10	12	22
Senior	15	16	31
Background/training			
Biomedical	11	12	23
Social science	14	16	30

Sampling was conducted by undertaking a systematic mapping exercise of Zambian and northern health researchers. Mapping included: researcher, institution, and project/collaboration, years of project/collaboration, topic, discipline(s) career stage. Initially, the intention of the authors was to identify a broad range of southern (Zambian) researchers who had participated in health policy and systems research partnerships, excluding clinical research collaborations. However, it became clear that most researchers who fell within this category identified their research as lying within the broad field of public health research. The primary inclusion criterion was public health researchers involved or recently involved (up to 5 years ago) in academic public health research involving Zambia and a northern country. Researchers were excluded where they had been involved in north–south health research studies that were operational for less than one year at the time of sampling, and where eligible research studies had been completed more than five years prior to the commencement of data collection for this study. The lead investigator sought to recruit a balance of senior (more experienced) and junior (less experienced) respondents. Hence, the respondents were selected based on their characteristics, in line with Given’s definition of purposive sampling [52]; and to obtain information and insights from those especially knowledgeable about or experienced with north-south partnerships [53].

Fourteen research collaborations were identified from the researchers selected. Sampling of researchers to interview from these collaborations included both a northern and southern representative from each of these collaborations. Six of these were classified as health policy and systems research collaborations, while 8 were in the broader field of public health, and 3 of which were Randomised Controlled Trials. Eight of these collaborations were multi-country research studies involving more than one northern and more than one southern country. Six were bilateral studies between Zambia and a northern country.

In-depth interviews were conducted between February and December 2011. Topic guides were developed which included researcher’s experiences of north–south health research at the various stages of the research process. Twenty six of the 28 Zambian interviews were conducted face to face, with 2 taking place over the phone. Five of the 25 northern interviews were conducted face to face and the remainder over the phone. Interviews were recorded and transcribed. Ethical approval was granted by the Royal College of Surgeons in Ireland Research Ethics Committee, where the lead author was working, and by the University of Zambia Humanities and Social Sciences Research Ethics Committee. Thematic analysis [54] was undertaken using *Nvivo 10*.

Reflexivity was central to the research process given the nature of the research, ie the primary researcher was analysing the field of north–south health research, but is also an actor (northern researcher) within the field. In keeping with the spirit of ethical north–south health research, a Zambian collaborator was invited to work with the northern researcher. The collaborator assisted with setting up the interviews, and provided contextual and cultural understanding.

## Results

### The field: north–south health research

A plethora of guidelines exist for north–south health research collaborations [4, 5, 46, 55, 56]. For the most part, these rules of research collaborations were broadly adhered to in the collaborations explored in this study, including for example the principles of honesty, accountability, professional courtesy, fairness and good stewardship, as outlined in the *Montreal Statement on Research Integrity* (2013). However, only one out of 14 health research collaborations in this study had developed ethical guidelines relating to partnership governance.

### Habitus of researchers

Bourdieu’s concept of *habitus* provides a valuable tool to gain insight into why north–south inequities and power imbalances exist and are perpetuated in north–south health research collaborations. Different *habituses* (experiences and expectations)*,* constituting a dichotomy between north and south, exist in north–south research partnerships. The legacy of colonialism was mentioned many times - equally by Zambian and northern researchers. Some northern and southern interviewees linked different work practices and approaches, by northern and southern researchers, to the culture of aid and colonisation. This association was sometimes attributed to Zambia having received considerable levels of aid contributing to the expectation that northern researchers would come to solve problems.

A number of Zambian researchers believed that Zambian researchers themselves had accepted these inequities and had chosen to work within the confines of the structure of what was presented to them. For example it was frequently mentioned that they accepted mid-level research positions, without striving for leadership positions, leaving these to their northern counterparts. Zambian respondents were frequently frustrated that so-called capacity building was in reality ‘*exposure’*. For some, this was considered to be patronising, as the form of capacity being developed was more about enabling Zambian researchers to collaborate better with northern researchers than developing capacity for independent country-level research:

*“I think one has to look at the history and say at what point and how do you do capacity building that’s not patronising in nature, and it’s not just simply about building up people’s capacity to collaborate better with Western researchers so that we get better data (north–south researcher 1).*

North–south inequities were verbalised throughout the interviews by northern and southern researchers, alike. Even when multiple identities of northern and southern researchers *and* a shared culture of research were mentioned by some interviewees, it was usually *in addition to*, rather than in the place of, north–south imbalances. It was clear that the northern/southern *habitus* was deeply rooted in these relationships. For example, in some situations, Zambian researchers considered it ‘*natural’* for northern researchers to set the agenda. There was often an assumption by Zambian researchers that research questions and data collection tools would be drafted in the north and adapted to the Zambian context, even in studies that were being undertaken in Zambia alone. This illustrates an acceptance of the *status quo*, in that this is how research partnerships have *always* operated.

Despite the acceptance of the *status quo*, many interviewees considered these imbalances to be unreasonable. Some examples were given by northern interviewees where opportunities to change the *status quo* had arisen for Zambian researchers: namely, opportunities to input into the research agenda, to lead data analysis and to lead on authorship of journal articles. However, some interviewees suggested that in many cases these opportunities had not yet been taken up, or that change was occurring at a slow pace.

Bourdieu’s *hysteresis* effect can provide a possible explanation for why this was the case. Perhaps these changes in north–south health research were subtle and the new emerging opportunities for Zambian researchers to lead had not yet been taken on board by many of them. There were also reports that Zambians often did not have their own research agenda prepared.

*“When you are constrained financially, I don’t know whether it is because of poverty, there comes a point where you stop thinking what you can do. … And so it is only when they actually see the money coming in that is when they start to think. We find computers with dust on them, we have vehicles that have been used but have not been used for any research. So in that sense you can blame us as the southern, the poor people, in not having enough of this stimulus on our own…” (Zambian researcher 5)*

However, some northern researchers noted that caution should be displayed against placing the onus on Zambian researchers to be the sole agents of change, instead recognising that northern researchers have a role to play in assisting to strengthen the capacity of Zambian researchers, to enable them to avail of these opportunities.

It was reported by some researchers—north and south—that some northern researchers have an interest in maintaining the *status quo*, in terms of maintaining control over the research process. However, findings show that northern researcher motivations in many cases are altruistic, in terms of improving health in Zambia, and capacity building for local researchers, or illustrate a combination of securing career opportunities.

A number of northern researchers explained that active attempts to address power imbalances and inequities were not successful. An example was given where a northern research institution played a non-interventionist role in one north–south health research collaboration, because they did not want to be seen as the dominant partner. However, this *laissez-faire* approach led to a perception that they were not pulling their weight on the project.

A number of northern researchers stated that the culture of the research institution in Zambia remains colonial, instilling in Zambians a culture of taking the back seat in north–south health research. In addition, some Zambian researchers accused northern researchers of continuing to carve up the country for research studies, with post-colonial connotations.

*“The country has been carved up by researchers and you stay in a guest house and you’re aware that XYZ universities in the US and UK are kind of ‘oh what are you working on?’ and there is almost a cautiousness about, oh well stepping on someone else’s feet, and that is so incredibly omnipresent in Zambia. And so there is this sense of too many researchers.” (northern researcher 13)*

### Capitals: north–south distribution

#### Economic capital

For the collaborations sampled, research funds flowed almost exclusively – with the exception of one study where funds were routed through a South African partner–through northern institutions, thereby instilling economic capital with the northern partners .^4^ Indeed, the primacy of economic capital was recognised by researchers themselves, many of whom concluded that as long as funding flows solely through the north, this will ensure that power remains with the north, no matter how much possession of other capitals shifts to either a state of equilibrium, or in favour of Zambian researchers.

Interviewees repeatedly cited research donors and northern researchers as occupying the dominant position in terms of dictating both the broad agenda and the partnership format, showing that being in possession of economic capital secures a knock-on effect for control in other elements of the research process. Many interviewees, north and south, pointed to the inability or failure of Zambian researchers to directly access research grants, and also noted that this was mirrored in Zambian research institutions, which – according to some respondents–lacked economic capital, through an unwillingness of donors to support them in building institutional capacity.

A common perception existed, by Zambian researchers, of poor individual Zambian researchers versus affluent individual northern researchers, and the oft repeated belief that Zambian researchers choose a research career only for the salary. It was frequently considered by Zambian researchers that choice due to actual interest in research or a particular research topic, was sometimes seen to be a northern luxury. One of the reasons given by Zambian researchers as to why research partnerships with the north should continue in the future was high northern economic status, which would provide opportunities for Zambians as well as northern researchers. It was also hinted by some northern researchers that they have too much to lose (i.e. their careers) by relinquishing control over funding.

*“We have to look at ourselves as Western researchers as to ‘what are we doing here?’ We can’t deny, as far as I’m concerned, the negative things that we are doing, and also what we need to give away. I mean if you’re actually looking for equity and balance, somebody’s got to give away something, and it’s pretty clear who has to give away stuff.” (northern researcher 5)*

Zambian researchers sometimes viewed that they *‘sell themselves’*, accepting northern initiated research partnerships in order to increase their economic capital, even though the agenda did not accord with Zambian priorities.

One perspective, reported by both Zambian and northern researchers, was that donors did not trust southern researchers to have the capacity to manage funds and account for the research budget, instead placing more trust in northern partners. It was considered by some northern and Zambian researchers that placing money in the hands of northern partners, gives donors an element of security that funding will be utilised in the most effective manner, making it easier to hold them to account for spending. This was described as important for the donors, *“to be assured that there is some amount of eyes and ears from northern partners, who provide comfort that the money will be used properly for the research that it was intended for” (Zambian researcher 3).*

Zambian Research Ethics Committees (RECs) were sometimes perceived by both northern and Zambian researchers to rubber stamp REC authorisation to obtain international funding. This was seen as unethical by some northern researchers.

#### Social capital

Social capital is particularly valuable in analysing researcher relationships in north–south research collaborations. While northern researchers generally had more direct access to social capital, due to connections with donors, both northern and southern partners mentioned being dependent on one another. This is due to donor requirements to include certain countries in a research bid, because the focus of the research is in the south, but also because the National Health Research Act (2013) stipulates that a Zambian must be a Principal Investigator or co-Principal Investigator on every study.

Examples were given by both southern and northern researchers that the latter used connections with Zambian researchers to steamroll agendas that northerners wished to pursue: *“They needed the African data to push their agenda forward. So in a way it was almost contract research. It was really their study idea but our study site”* (north–south researcher 23). However some Zambian researchers considered that the onus is partly on Zambian researchers, to seek out northern researchers who have an interest in health research topics of relevance in Zambia. However, eligibility for funding is often pre-defined, where northern researchers have ring fenced access to their own national or EU research funding for pre-defined topics, and is therefore outside the control of Zambian researchers. A number of Zambian interviewees stated that Zambian researchers *could* set the agenda, and that they themselves only accepted a proposal if it matched Zambian priorities.

A perspective that strongly emerged from northern and southern researchers was that even where there are positive social connections between researchers, such as trust and respect, north–south inequities and power imbalances continued to exist due to imbalances in economic capital.

#### Symbolic and scientific capital

The perception existed by many northern and Zambian interviewees, that Zambian research institutions did not have the capacity to manage funds and were seen by donors to be risky. This was considered to be in contrast with northern researchers/ institutions possessing the prestige to be viewed by funders as trustworthy. This could be attributed to the reputation—or symbolic capital and lack of it—which both north and Zambians have acquired over time. It could also be based on genuine reasons for considering Zambian researchers (or their institutions) as risky.

There were examples cited of scientific capital at play. In one case it was reported that two northern researchers had pursued a particular research topic, despite the Zambian Ministry of Health stating that it was not relevant to the needs of the country. This suggests that scientific capital or *‘academic power’* [57] stemmed from or was reinforced by the economic capital of the northern researcher. Other examples included northern researchers reporting a general Zambian unwillingness to input into drafts of research proposals. This could signify an absence of ownership of the research; and/or a lack of capacity to input, both of which can be interpreted as less (or less confidence in their) scientific capital.

One South African researcher reported being ‘*forced’* to undertake research analyses, i.e. did so reluctantly, even when she had not been involved in the design of the study and lacked knowledge of the Zambian health system. Through an analysis of Bourdieu, this can be attributed to symbolic/scientific capital of northern and South African researchers, through an assumption that their capacity will be higher.

*“I had no input in the research design, how the questionnaires were developed, how the research was conducted, and then this mythical idea that I could come in and I could just look at the data and write-up, so really it was completely ridiculous. It still perplexes me as to how people thought that I could just look at data and come in, having not been at all involved, and from South Africa.” (north–south researcher 17)*

Published literature reveals that most articles relating to LMIC contain authors from HIC and also that the first author is likely to be from a HIC [10, 58, 59]. This study found evidence on inequities in authorship of papers from northern and southern perspectives. Despite some interviewees indicating that the process was seen as equitable and ethical with both northern and Zambian researchers having equal opportunities to publish, several experiences of authorship being controversial were communicated. Many northern interviewees reported a bias in favour of including Zambian collaborators, claiming that even though northerners undertook most of the writing, they could not put themselves as first author. Some researchers, north and south, saw this as being unethical, whereas other perceived it as being important in terms of visibility and building capacity for southern researchers.

*“So now I’m trying to submit a paper on his behalf, he’s the first author. I can’t even get hold of him to get him to agree that it’s ok, it can go in. But ultimately if there is a problem with it he’s the first author. And I’m not sure what the ethics of…should I submit a paper with somebody else’s name first? But if I put my name first that would be wrong.... It doesn’t seem quite right but I suppose maybe that’s the way these collaborations do work in the end.” (northern researcher 8)*

However, other researchers stated that it is the norm for the first and senior author to be non-Zambians and for the Zambians to be in the middle.

Most respondents discussed research capacity strengthening from a one-way, north-south perspective, suggesting that due to the symbolic (scientific) capital of northern researchers, there is an assumption that they alone have the knowledge and capacity to impart to the south.

#### Cultural capital

Cultural capital plays a major role in research partnerships that transcend cultural and geographic divides. Despite interviewees’ views that northern researchers lacked understanding of context in agenda setting and research design, a number of Zambian interviewees recounted situations where northern researchers insisted on standardised questions, even though they were not well suited to the Zambian context.

A number of northern and Zambian researchers mentioned that the culture of the Zambian research institution is colonial (institutionalised cultural capital), which instils in Zambians the practice of taking a back seat in health research. Both Zambian and northern researchers described that northern researchers lead on data analysis, even though in most cases they did not lead on the data collection process and were often perceived to have a poor understanding of Zambian contextual issues. Some northern researchers were acutely aware of this, expressing their discomfort with the situation. However, other northern researchers did not see this deficiency in cultural awareness as presenting a problem. This suggests that symbolic/scientific capital of northern researchers can override the cultural capital of those in the south, through their assumption that they have greater capacity to undertake data analysis.

At the point of fieldwork, Zambian respondents often viewed northern researchers as lacking the capacity to understand the culture and context of communities, particularly those who participated in fieldwork for only short periods of time. Northern researchers were often aware that their presence could have an influence on the research, such as an expectation that participation in research would result in an increase in health services. Many Zambian researchers also shared this view.

*“They will say ‘oh here is a muzungu, this woman what does she want, let’s tell her what will make her happy’. So much of the time the way they will answer will not be because of what they think, but they will say what is it that she wants to be happy with. So they will try to please you.” (Zambian researcher 14)*

Some northern respondents felt that it was important that they take part in some of the data collection - recognising the need to acquire some degree of cultural capital. A number of Zambian interviewees contended that Zambian researchers need to be “*custodians of our own culture” (Zambian researcher 21),* suggesting they should have an innate closeness with the communities being researched, even when they were not considered to be complete insiders, and sometimes quite distanced from the communities of study.

*“Sometimes…I am corrupted too. When I was growing up… I knew my own language very well. But then as you grow up and you begin to go into English… I lose track… But somehow I believe I am one of them. But I need to respect and understand my people better because I have been out for a long time. We as educated Zambian researchers should understand that we have gaps in trying to reach and understand our own communities as well.”* (Zambian researcher 17)

## Discussion

While a number of researchers spoke of trust and equity amongst partners, many perceived relationships and roles to be inequitable with power remaining in the north. An analysis of findings has shown that northern researchers were more likely than Zambian researchers to consider power imbalances as ethical issues, with Zambian researchers more likely to consider as ethical issues, traditional research ethical concerns, such as research ethics review processes and informed consent.

This study highlights the perspectives of one set of actors—researchers—in respect to the different dimensions and different distributions of powers in research collaborations, through concepts from Bourdieu’s theory of *Power and Practice*. Though not considered in this paper, it is recognised that other actors, such as research donors, research participants, communities and policy makers are also key players to be considered. Furthermore, dissemination and getting research into policy and practice stages of the research process have not been included, which would be desirable to gain a complete picture of power across each stage of the research process. Both of these were beyond the scope of the study conducted.

The application of concepts from Bourdieu’s theory of *Power and Practice* [38] has a number of uses for highlighting new aspects of research ethics at different stages of the research process. Firstly, *habitus* helps us to understand the sometimes unconscious maintenance of the *status-quo* in north–south health research, which most southern and many northern researchers see as inequitable. This article illustrates that the views of both sets of researchers support the conclusion that different *habituses* exist, north and south, which are central to understanding the dynamics and ethics of north–south health research. These are *habituses* of domination (northern) and subordination (Zambian) in relation to researcher relationships. It is recognised that these are generalisations and stereotypes, used for typing and analysis of findings. However, the results showed a propensity among most respondents from each location to gravitate towards these states.

Secondly, Bourdieu’s *hysteresis* effect provides a possible explanation for why power differentials continue to exist. In some cases, new opportunities have arisen for Zambian researchers; however, they may not immediately recognise and grasp them [44]. Perhaps these changes in north–south health research are subtle and the increasing opportunities for Zambian researchers (in agenda setting, data analysis and authorship) have not yet been fully recognised or taken on board by the Zambian researchers. Studies in the area of north–south health research consistently report a lack of capacity in the south as being one of the major reasons for north–south power imbalances [41, 42]. It may also be the case that research donor claims that they wish to see southern-driven research has been lip-service, not supported by institutional capacity-building action. If this is the case, new opportunities without sufficient capacity strengthening will result in the maintenance of the *status quo*.

Thirdly, Bourdieu’s’ concept of *Capitals* allows us to explain how diverse resources are used to explain these power imbalances, where northern researchers are often in possession of more economic, symbolic and social capital *vis-a-vis* Zambian researchers; while Zambian researchers possess more cultural capital *vis-a-vis* northern researchers. Literature in the area of international health research collaborations illustrates that while power is regularly reported in north–south health research [6, 7, 18, 25], it is rarely discussed in detail between research partners. Recognition of the different dimensions and fields of power that are occupied and exercised by northern and southern researchers could pave the way for more equitable partnerships. For example, by explicitly recognising and valuing the different forms of capital, greater weight would be given to non-economic capital and recognition of the contribution of all partners. This could help not only achieve greater north–south equity, but could also lead to more rigorous and more culturally contextualised research. This paper therefore encourages the scope of ethical reflection to be broadened to consider the broader situated ethics of north–south health research, which “*takes into account the realities of complex individual, institutional and national imbalances in power and resources.”* [11]

Bourdieu’s work has been criticised for concentrating on the internal analysis of fields, which may encourage a loss of sight as to how fields are connected into broader society; and his framework stresses the propensity to perpetuate structures inherited from the past, rather than encouraging researchers to seek out forms of change [57]. This study has used Bourdieu’s analytical framework to explore and understand the views, actions and non-actions of researchers interviewed rather than to understand how the situation could change.

## Conclusions

This article has highlighted the importance of considering as ethical issues, not just traditional notions of research ethics, such as research ethics review processes but also macro research ethics, of which power is a central component of international public health research involving the global north and the global south.

The argument that power imbalances need to be redressed is based on an assumption that research partnerships should at the very least balance knowledge, interest and power in the short term, with the aim of maximising benefits for LMICs in the longer term. Many of the imbalances are structural, rather than within the realms of researcher relationships. This means that even if relationships can be altered, structural inequities will remain dominant (for example economic capital in the form of research funding), thereby ‘trumping’ symbolic, social or cultural capital. Addressing relationship inequality is often constrained by inequalities at the structural level, which is within the hands of other actors in the research process, such as donors.

To date, most studies of north–south health research collaborations have been self-evaluations. While this in itself is positive, often authors do not state their positionality in relation to the research. Therefore, it is important to explicitly acknowledge that the three authors of this paper are all northern (Irish) public health / health systems researchers, even if two of them have lived and worked for six and twenty years, respectively, in sub-Saharan Africa and the other has worked and visited Zambia as part of one research partnership. The use of concepts from Bourdieu’s theory of *Power and Practice* could be used to explain power differentials in other forms of partnership between northern and other southern countries. The findings in this paper support the view that the future for north-south health research is one of co-dependency, at least in the short to medium term; and there was a consensus among northern and southern researchers, broadly speaking, that the mutual benefits outweighed the disadvantages of north–south collaborations. However, inequities and power imbalances need to be recognised and addressed and the situated ethics approach, taken in this paper, needs to be seen as a central ethical concern in public health research.

This concept of situated ethics has potential relevance and application to southern Africa, for example in understanding power differentials in research relationships between southern Africa and HIC. A recognition of the different elements of power by northern and southern researchers could pave the way for more equitable partnerships. This can be achieved through recognising the importance of considering as ethical issues, not just traditional notions of research ethics, but also a situated research ethics, as described in this paper. Considering only one of the partnerships in this study had developed research ethics guidelines relating to the partnerships themselves, it is recommended that research partnerships incorporate a situated ethics approach by developing ethical guidelines relating to health research partnership governance and for operationalising these*, or;* contextually adapting and utilising existing health research partnership guidelines such as the Swiss Commission KFPE Guide for Transboundary Research Partnerships (2012) [4]

## Abbreviations

HIC: High income country
LMIC: Low and middle income country
REC: Research Ethics Committee

## Footnotes

1 A systematic search process identified 8,000 potential references, 239 of which fitted the inclusion criteria. An abundance of commentaries and debates exist, and where empirical research exists, it is overwhelmingly in the form of self-reflection and self-evaluation which usually focus on one aspect of the research partnership or research process, for example research capacity strengthening or authorship in north–south health research. Ninety-four empirical studies were identified, 57 relating to macro research issues, and 37 relating to micro research ethical issues. The literature review identified 13 evaluations of north–south health research, of which 9 were self-evaluations. Following piloting of search terms relating broadly to north–south health research, 4 databases were systematically searched: Web of Knowledge, Pubmed, Global Health, and *Scirus*. The following search terms/strings were used: (health OR medical OR medicine OR biomedical OR biomedicine OR clinical OR “tropical medicine”) AND (partner* OR network* OR consort* OR collaborate* OR alliance* OR cooperate* OR co-operate*) AND (research) AND ((global OR international OR transnational OR multi-country OR (multi AND country) OR cross-country OR (cross AND country) OR (north AND south)) OR (“developing country” OR “developing countries”) OR ((lower OR middle) AND income)). The following inclusion criteria were applied.

1. Research on: human health/ (bio)medicine/clinical/tropical disease.
2. Research to involve at least two countries.
3. At least one of the partners must be a LMIC (any region).
4. Academic research: researchers working in Universities/research institutions (for example community/policy partnerships not included).
5. The project/collaboration process must be detailed, rather than an exclusive focus on results of the research.

2 The literature review identified 13 evaluations of north–south health research, of which 9 were self-evaluations.

3 The South African researcher was placed in the ‘north-south’ category. South African researchers are often considered to be northern by other African researchers, but considered to be southern by most northern researchers.

4 It should be noted that other models of funding exist, where funding is channelled directly south, such as the Alliance for Health Policy and Systems Research. This model was not represented within this study sample.
